# Mining for Active Molecules in Probiotic Supernatant by Combining Non-Targeted Metabolomics and Immunoregulation Testing

**DOI:** 10.3390/metabo12010035

**Published:** 2022-01-04

**Authors:** Juliano Roldan Fonseca, Marianna Lucio, Mourad Harir, Philippe Schmitt-Kopplin

**Affiliations:** 1 Research Unit Analytical BioGeoChemistry, Helmholtz Zentrum München, 85764 Neuherberg, Germany; jurfonseca@gmail.com (J.R.F.); mourad.harir@helmholtz-muenchen.de (M.H.); schmitt-kopplin@helmholtz-muenchen.de (P.S.-K.); 2 Analytical Food Chemistry, Technical University of Munich, 85354 Freising, Germany

**Keywords:** probiotics, solid-phase extraction, ESI[±] FT-ICR-MS, untargeted metabolomics, metabolic footprinting, cell culture supernatant, tryptophan pathway, bioactive compounds

## Abstract

Chronic respiratory diseases such as asthma are highly prevalent in industrialized countries. As cases are expected to rise, there is a growing demand for alternative therapies. Our recent research on the potential benefits of probiotics suggests that they could prevent and reduce the symptoms of many diseases by modulating the host immune system with secreted metabolites. This article presents the first steps of the research that led us to identify the immunoregulatory bioactivity of the amino acid d-Trp reported in our previous study. Here we analyzed the cell culture metabolic footprinting of 25 commercially available probiotic strains to associate metabolic pathway activity information with their respective immune modulatory activity observed in vitro. Crude probiotic supernatant samples were processed in three different ways prior to untargeted analysis in positive and negative ionization mode by direct infusion ESI-FT-ICR-MS: protein precipitation and solid phase extraction (SPE) using HLB and CN-E sorbent cartridges. The data obtained were submitted to multivariate statistical analyses to distinguish supernatant samples into the bioactive and non-bioactive group. Pathway analysis using discriminant molecular features showed an overrepresentation of the tryptophan metabolic pathway for the bioactive supernatant class, suggesting that molecules taking part in that pathway may be involved in the immunomodulatory activity observed in vitro. This work showcases the potential of metabolomics to drive product development and novel bioactive compound discovery out of complex biological samples in a top-down manner.

## 1. Introduction

The hygiene hypothesis suggests that the increase of atopic diseases in the modern civilization (e.g., asthma, allergic rhinitis, and atopic dermatitis) may be caused by the decreased exposure to microbes during the children’s first year of life, particularly in developed countries and urban areas. High hygiene standards, use of antibiotics, less breastfeeding, and processed-food diets may prevent the normal development of a baby’s immune system by not challenging it to respond to different threats during its maturation [1,2]. A comparable theory was proposed by Metchnikoff at the beginning of the twentieth century, which is considered to be the precursor of the concept of probiotic bacteria. He suggested that the Bulgarian rural population had a longer and healthier life span due to their high consumption of fermented milk containing the bacterium “Bulgarian Bacillus”, today called *Lactobacillus bulgaricus* [3]. However, it was only in the mid-1990s that “probiotics” received proper attention from the medical community as potential therapeutic agents [4]. Today, probiotics represent a serious research field where health claims have been made on a variety of diseases and a multibillion dollar global market has emerged around it, full of popular products that are now part of our daily life [5].

Atopic diseases occur due an exaggerated and unbalanced immune response to environmental or food allergens and their treatment is based on interventions with antihistamines, glucocorticoids, steroids, or bronchodilators that provide relief of symptoms but no cure [6]. In this context, probiotics have been studied as a therapeutic alternative. *Lacticaseibacillus rhamnosus* GG (LGG) was given to pregnant women prior to delivery who had at least one first-degree relative with atopic eczema, allergic rhinitis, or asthma, and then to the newborns for six months. The results showed the frequency of atopic eczema in neonates was lower in the probiotic-treated group than in the placebo-treated control group [7]. The use of the strains *Bifidobacterium lactis* BB-12 and LGG to control allergic inflammation was studied in 27 newborns that manifested atopic eczema during breast-feeding and an improvement in skin condition after two months in all the infants receiving probiotics was reported, supported by a decrease of soluble CD4 glycoprotein concentration in serum (a marker of T-cell activation) and eosinophil protein X in urine (a marker for inflammatory activity) [8]. Similar results were observed in another clinical trial where combination of probiotic strains was administered for eight weeks to 40 children with atopic dermatitis. The total Immunoglobulin E (IgE) level in serum decreased for the probiotic treated group; an antibody that triggers “immediate hypersensitivity” reactions [9]. The called “PandA study” reported a reduced incidence of infant eczema in the probiotic-administered group (six weeks prenatally to mothers and 12 months postnatally to offspring) with positive effects persisting through one and two years. Reduction of cytokines that regulate many aspects of allergic inflammation was observed in whole blood cells obtained from probiotic-supplemented infants [10].

Nevertheless, inconsistency in clinical outcome has challenged the role of probiotics in allergy prevention. Two reviews articles have drawn attention to the lack of robustness of several published studies challenging the positive results observed after administration of probiotics to infants for the prevention of allergic disease [11,12]. The Food and Drug Administration (FDA) has defined probiotics as live biotherapeutic products and requires sufficient clinical investigation following the principles of drug-like development [13] (p. 10). The European Food Safety Authority has issued unfavorable opinions on health claims for probiotic and one of its major reasons was the lack of information on the substances causing the effect on which the claim is founded [14].

Besides the efforts of the scientific community, the interaction between probiotics and host remains only partially understood. The controversial results from probiotics research show how complex molecular crosstalk could be between probiotic bacteria, the gut microflora, and the immune system of the host. The ability of probiotics to regulate host immune system using several bacteria derived genes and proteins have been described [15]. Our own commensal bacteria also regulate immune responses beyond gut environment. It is suggested that symbiotic bacteria-derived metabolites such as carbohydrate-binding proteins, short-chain fatty acids, long-chain fatty acids, and biogenic amines might affect host immune maturation, activation, and functions leading to tolerance or pro-inflammatory responses [16,17,18].

Studies on microbiome-related treatments for immunological disorders using supplemented microorganisms are still needed. However, rational selection of the probiotic strain(s) that should be subjected to non-clinical or clinical trials based on the potential bioactivity of their derived metabolites, is not a common practice so far.

Bioassay-guided fractionation has been the traditional discovery approach to new bioactive compounds. The work starts with a complex crude extract from a source of potential bioactive chemical entities (i.e., plants, fungi, marine organisms, microorganism cell cultures, etc.), and components are separated into fractions based on compounds physicochemical properties using different techniques such as chromatography, liquid-liquid extraction (LLE), supercritical fluid extraction (SFE), solid phase extraction (SPE), distillation, among others. An in vitro biological system (bioassay) tests for the effect caused by each fraction. Alternating between further fractionation and bioassay responses will likely lead to the isolation and identification of the compound/s with biological activity [19]. It is a time-consuming process and sometimes the bioactive effect of a fraction is lost after successive fractionation steps due to the dilution of bioactive components to an inactive level, lack of long-term stability of components, or lack of synergistic activity of more than one component once each of them are separated into different fractions, for example.

**Figure 1:**
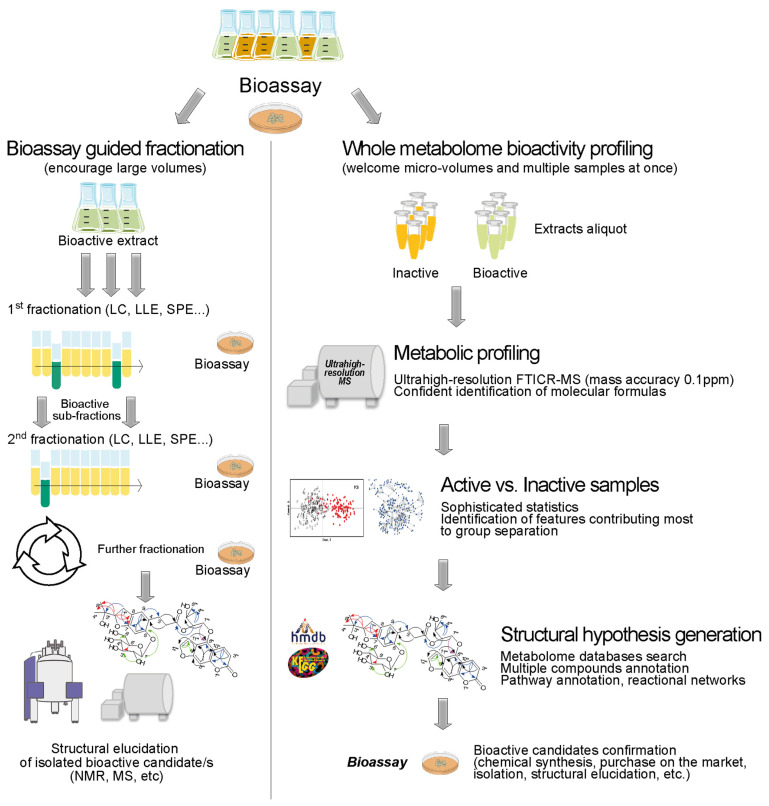
Schematic representation of two approaches to new bioactive compound discovery. (**Left panel**) Traditional bioassay-guided fractionation is time and labor intensive due to its step-by-step separation using chromatographic techniques followed by biological activity assessments. It removes most of the interfering matrix compounds but may lead to losses of active compounds. (**Right panel**) Holistic metabolomics approach deals with complexity, requires high level of expertise in instrumentation and data analysis, but offers scientists a broader picture of the biological system in study [19].

Enormous molecular diversity and biological functionality are two important features that metabolomics can cover at once, revealing potential bioactive compounds or at least leading to hypothesis that will set the ground for further investigations. We recently applied targeted and non-targeted metabolomics strategies based on mass spectrometry in a clinical intervention trial with probiotics [20]. Metabolomics was shown to be a valuable approach to the discover of new bioactive molecules in biosamples [21,22,23,24], and out of extremely complex organic environmental samples [25,26]. In vitro, metabolomics can provide important information on cell-to-cell communication, on cell growth behavior, on metabolite flux supporting metabolic engineering and industrial biotechnological processes, and has been successfully applied to study bacterial culture supernatants [27,28,29,30]. Figure 1 shows the workflow differences between the classical bioassay-guided fractionation and the metabolomics approach in the discovery of new bioactive compounds.

Here we want to highlight the first research steps we followed before our discovery of D-tryptophan as a modulator of the gut microbiome and allergic airway disease [31,32]. This study presents our rapid screening approach via direct infusion of 25 samples derived from probiotic supernatant in high-resolution Fourier-transform ion cyclotron resonance mass spectrometry (FT-ICR-MS), with the objective to simultaneously screen as many metabolites as possible, and to discriminate supernatants that showed immunomodulatory properties from inactive ones by applying multivariate statistical analysis to the acquired data. For that, aliquots of each probiotic culture supernatant were subjected in parallel to three different sample cleaning procedures prior to FT-ICR-MS analysis: A simple protein precipitation from the crude supernatant and solid-phase extraction (SPE) with the HLB hydrophilic polymeric phase and cyano-propyl sorbent (CN-E) phase (i.e., each strain supernatant generated three samples (Crude Supernatant, HLB-SPE extract and CN-E-SPE extract) that were analyzed after electrospray ionization (ESI) in positive and negative modes).

## 2. Results

### 2.1. Probiotic Strains and Their Respective Supernatant Immunomodulatory Response

Each individual probiotic culture supernatant was harvested at early stationary phase since bacteria are at their maximum size and cell density and, in theory, have maximized production of metabolites [33]. These supernatants previously showed a concordant immune response in two different bioassay screenings [31] (i.e., it downmodulated costimulatory molecules (bioactive) or it was inactivity). The outcome was not species-dependent (Table 1).

### 2.2. FT-ICR-MS Analysis and Data Processing Prior Statistics

Prior FT-ICR-MS analysis, each individual probiotic supernatant was subjected to three different desalting extraction procedures to generate three organic extracts using methanol: two after solid phase extraction (HLB-SPE and CN-E SPE) and one after a simple protein precipitation of crude supernatant. These extracts also correspond to differential chromatographic fractionations leading to chemical mixtures with various biological responses. Analysis of each extract by FT-ICR-MS in both electrospray ionization (ESI) modes (positive and negative) resulted in an ASCII file containing the mass to charge (*m*/*z*) and their respective signal abundance. To investigate differences in the metabolome data between bioactive and non-bioactive probiotic supernatants, individual sample ASCII data files were combined according to sample type and ESI mode resulting in six data matrix: crude supernatant/ESI[+], crude supernatant/ESI[-], HLB-SPE extract/ESI[+], HLB-SPE extract/ESI[-], CN-E-SPE extract/ESI[+], and CN-E-SPE extract/ESI[-]. After mass signal alignment, mass filtering and molecular formula assignment, each data matrix showed several molecular features. The highest number of features was observed for the data matrix HLB-SPE extract followed by the matrix CN-E-SPE extract, both in ESI negative mode (Table 2).

### 2.3. Principal Component Analysis and Orthogonal Partial Least Square Discriminative Analysis

**Figure 2:**
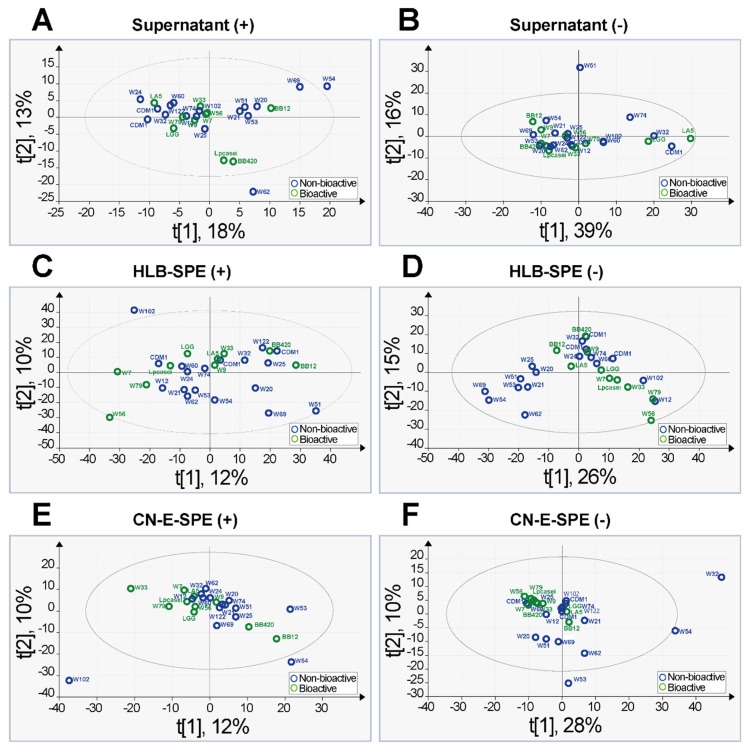
Unsupervised PCA scores plots obtained from the data matrix of samples of crude probiotic supernatants (**A**,**B**), HLB-SPE extracts (**C**,**D**), and CN-E-SPE extracts (**E**,**F**). The left side displays results obtained from FT-ICR-MS analyses in ESI positive mode and the right side in ESI negative mode. Only models D and F showed a group separation trend with predictive relevance.

Unsupervised principal component analysis (PCA) was applied to each individual data matrix without any input on sample classification to the model. The score plots obtained from these analyses are shown in Figure 2. Only two models showed a group separation trend (Figure 2D,F). The data obtained in ESI negative ionization mode for samples submitted to CN-E-SPE extraction showed a more homogenous grouping of bioactive supernatants when compared to other models suggesting that this model has predictive relevance. The strong homogeneity between the bioactive groups in that model is underlined through the presence of some outliers (samples from W32, W54, and W53). This suggests a more unique character of these inactive samples. Data matrix PCA analysis for HLB-SPE extracts/ESI[-] (model D) describes a particular pattern on correlated samples belonging to the group of non-bioactive supernatants in the lower-left quadrant. Samples in this cluster were originated from cell culture of different probiotic species (Bifidobacterium, Lactobacillus, Streptococcus, and Enterococcus), but those samples may share some similarities in their detected metabolic profile. This model also suggests predictive relevance.

**Figure 3:**
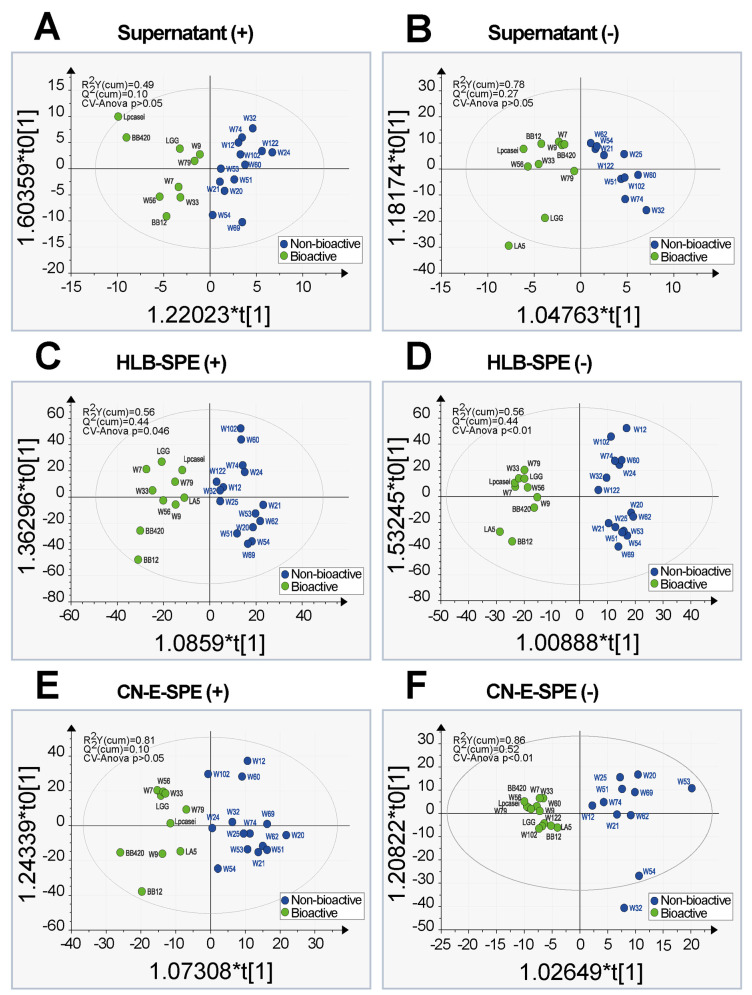
Supervised OPLS-DA scores plots. Models C, D and F are robust and have a high classi-fication power. Discriminant molecular features (*m*/*z*) observed in these three models were submitted to metabolic pathway analysis. The left side displays results obtained from FT-ICR-MS analyses in ESI positive mode (**A**,**C**,**E**) and the right side in ESI negative mode (**B**,**D**,**F**).

Figure 3 shows the OPLS-DA score scatter plots for the six calculated models after seven-fold cross validation. The values that indicate the goodness of the fit (R^2^) and the different levels of predictability (Q^2^) are reported in the plots. We also added the *p*-values referred to the CV-ANOVA. We could achieve for the models C, D and F a Q^2^ value closer to 0.5. This could suggest that the model has predictive relevance for a particular reflective endogenous latent variable (Q^2^ = 0.5 indicates a good model; a threshold of 0.5 is generally admitted for metabolomics [34]). Highly divergent values of R^2^ and Q^2^ values would indicate model over fitting (i.e., poor prediction power) [35,36]. Examining the results, we could infer that only three out of six models had a robust classification power: model F (CN-E-SPE extracts/ESI[-]), which showed already a separation of groups in unsupervised PCA even though three inactive samples were misclassified to the bioactive class (W60, W102 and W122); model D (HLB-SPE extract/ESI[-]) with its high classification power without misclassification of samples; and model C (HLB-SPE extract/ESI[+]) which showed also a good classification power although CV-ANOVA *p*-value was higher for this model (*p* = 0.046) than for the models D and F.

### 2.4. Metabolic Pathway Assessment

**Figure 4:**
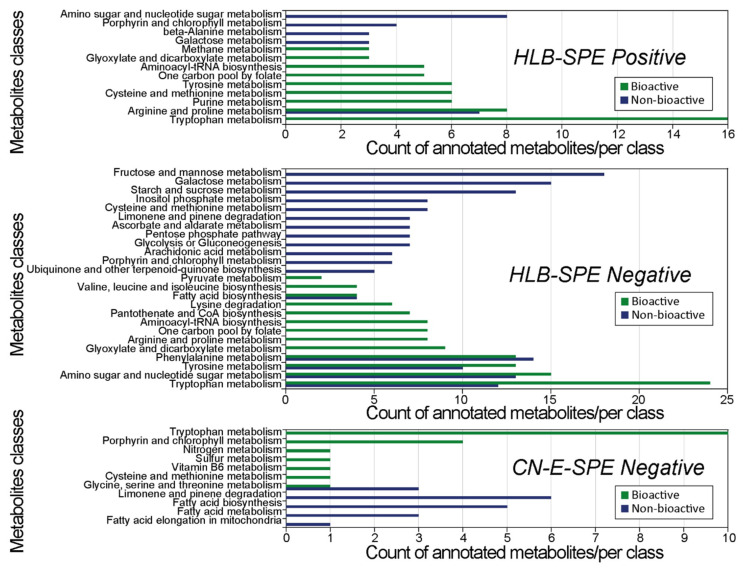
Description of the most populated KEGG metabolic pathways and their respective number of hits. Pathway analysis was originated from the most discriminant mass features of each class derived from the OPLS-DA models that showed robust classification power.

The most discriminant *m*/*z* features revealed by OPLS-DA for each class in all robust models (Figure 3C,D,F) were submitted to MetaboAnalyst web-based platform for metabolic pathway analysis using the “MS Peaks to Pathways” software feature; specially designed for untargeted metabolomics [37]. As no library of bacteria species was available in MetaboAnalyst by the time of this assessment, *Homo sapiens* was chosen as specie to perform pathway analysis since it has the most comprehensive organism library in the KEGG metabolic network database [38,39]. The resulting pathways activity listed by MetaboAnalyst was compared to the reference pathways represented in the KEGG database for species of bacteria analogous to those investigated in this study. Only bacteria pathways listed in KEGG which were the most populated with hits, and pathways reflecting the conditions of the experiment were considered for the data interpretation (e.g., nicotine and caffeine metabolism were excluded). Figure 4 shows that unique pathways were populated for each class of supernatants in the three models, with little overlap between the two classes and between the results of the three data sets.

### 2.5. Tryptophan Metabolism

Remarkably, the tryptophan (Trp) metabolic pathway was overrepresented in the bioactive supernatant class in all three models. Especially in the HLB-SPE extract/ESI[+] and CN-E-SPE extract/ESI[-], no hits related to Trp metabolism were observed for the non-bioactive class. Metabolite hits predicted in the Trp pathway were compared between models and classes (Table 3). A comparison visualization using Venn diagrams is shown in Figure S1. In fact, only 16 hits were obtained for the dataset HLB-SPE extract/ESI[+], but no unique one. A total of 24 hits were obtained for the dataset HLB-SPE extract/ESI[-], where seven hits were unique. Ten hits were obtained for the dataset CN-E-SPE extract/ESI[-], being one hit unique. A total of 25 different hits related to tryptophan metabolism was counted for the class of bioactive supernatants combined. Discriminant features for the non-bioactive class of supernatants showed only three unique hits related to Trp metabolism, while nine hits were shared with the bioactive class. Out of these nine shared hits, six were present in all evaluated data matrices, two were present in HLB-SPE extracts but not in the CN-E extract, and one was only present in the HLB-SPE/ESI[-] matrix. Trp amino acid was annotated as discriminant feature only in the bioactive supernatant data set of HLB-SPE extracts in both positive and negative ionization mode.

## 3. Discussion

### 3.1. The Approach to Sample Generation and Untargeted Metabolomics

The pretreatment of biological samples is usually required prior instrumental analysis to reduce matrix complexity and remove salts. The number of *m*/*z* features present in each data matrix reflects that (Table 2). Solid-phase extraction was more effective in producing a cleaner sample than a simple protein precipitation, lowering baseline noise and improving MS detection. The influence of different sample extraction techniques and different electrospray ionization mode on the chemical diversity detected by FT-ICR-MS was also demonstrated, even if pathway activity assessment was performed on statically discriminant *m*/*z* features only (Figure 4). Based on our experience and on information provided by SPE suppliers, the HLB-SPE and CN-E SPE extraction was chosen in an attempt to cover a significant wide range of metabolite classes [40,41]. Due to its non-polar and polar functional groups (the hydrophilic N vinylpyrrolidone and the lipophilic divinylbenzene), the HLB hydrophilic polymeric phase can retain a much broader range of polar to non-polar, neutral and acidic to basic compounds than traditional reversed-phase C18-silica-based SPEs. HLB phases are also very resistant to over-drying, making extraction methods more reproducible and robust [42]. As a supplementary phase, the medium polar cyano propyl sorbent (CN-E) can extract from aqueous matrices moderately polar to non-polar compounds that would be irreversibly retained on non-polar sorbents such as C8- and C18-based phases.

Overlapping of discriminant metabolic pathways and metabolite hits were observed between different data matrices, as shown in Table 3 and Figure 4. The shared hits between the bioactive and non-bioactive class in the tryptophan metabolism pathway may be explained by the presence of structural isomers and stereoisomers (e.g., N-Acetylindoxyl, Indole-3-acetic acid and 3-Indoleglycolaldehyde (C10H9NO2); Indolelactate and 5-Methoxyindoleacetate (C11H11NO3); l/d-Formylkynurenine and 5-Hydroxy-l/d-tryptophan). We believe that predicted metabolites shared between both classes of samples still deserves an activity test in bioassays. Additionally, it is expected that some metabolites are retained by both HLB and CN-E solid phases and/or can be ionized in ESI positive and negative modes, and we acknowledge the redundancy potential in the data obtained from a single probiotic supernatant that was subjected to these different techniques. However, each individual supernatant extract originated a data set that was combined into a data matrix, and some metabolite hits were predicted across different matrices for the group of bioactive supernatants only (tryptophan, indole pyruvic, serotonin, etc.). Results of a single data matrix validate the ones of other matrices as each individual probiotic sample validates the group (bioactive or non-bioactive), reinforcing hypothesis towards potential bioactive metabolites. Yet, it should be noted that the data generated from both SPE fractions and ionizations modes clearly differed from each other and this piece of information can be useful to drive the isolation and identification of a specific class of metabolites by using the proper extraction technique. We did not want to miss that by combining different data sets prior data processing and statistics.

### 3.2. Immunomodulatory Supernatants and Bioactive Pathways

The results of pathway analysis suggest that tryptophan related metabolites produced by probiotic bacteria may be involved in the underlying biological processes of immunomodulation observed in our in vitro test systems. This study was followed by another one where bioassay-guided fractionation was applied to the two most bioactive supernatants and structural elucidation using instrumental analysis (enantiomeric chromatography separation, NMR and FT-ICR-MS) revealed D-tryptophan has been the immunomodulator compound present in a bioactive fraction, which was confirmed in in vitro and in vivo experiments [31]. In this case, the classical strategy validated the hypothesis originated with our high-throughput nontargeted FT-ICR-MS-based metabolomics towards the importance of tryptophan metabolic pathway to immunomodulation.

In vivo, the balance between activation and suppression of the immune response depends on various regulatory mechanisms, where the impact of microbiome and Trp metabolism on immune tolerance has been much investigated [43,44]. Two enzymes are well known to drive the first step of the amino acid Trp into the kynurenine pathway. In the human body, the rate-limiting enzyme Trp 2,3-dioxygenase (TDO) converts l-Trp into N-formyl-l-kynurenine in the liver, while indoleamine 2,3-dioxygenase (IDO) does the same but is primarily expressed in epithelial cells, stem cells, and cells of the immune system such as monocytes, macrophage, and dendritic cells (DCs). Thus, IDO has been studied and recognized to have immunoregulatory roles [45,46]. Overexpression of IDO was correlated with immunosuppression and tolerance in vitro and in animal models, likely, due to the depletion of tryptophan and production of bioactive metabolites such as kynurenine, 3-hydroxy-kynurenine, or xanthurenic acid which seems to suppress T-cell responses, induces regulatory T cells and facilitates the development of regulatory DCs [47,48,49]. However, the effect of Trp degradation on human DCs is still unclear. For instance, it has been demonstrated in vitro that human DCs increased the expression of inhibitory receptors and showed significantly lower stimulatory capacity toward T cells under low Trp concentration conditions, but no difference on the stimulatory capacity was observed when a mixture of the metabolites anthranilic acid, 3-hydroxykynurenine, 3-hydroxyanthranilic acid, and quinolinic acid was added a prior to the medium; contrarily of what was observed for T-cells [50]. On the other hand, Belladonna et al. showed that nearby produced kynurenines and derived metabolites can be taken up by murine DCs inducing a tolerogenic phenotype, independently of Trp availability and IDO activity, i.e., IDO-competent cells can “transfer” tolerogenic potential to DCs lacking functional IDO by producing Trp metabolites [51]. Manni et al. confirmed those findings showing that exogenous l-kynurenine induced endotoxin tolerance on mice DCs that were submitted to a lipopolysaccharide (LPS) induced hyperresponsiveness [52]. Together with Trp metabolism, immunomodulation related to L-arginine metabolism (also well populated in the bioactive group of supernatants) has been well described in the literature [53]. Co-expression and co-activity of both IDO and Arginase 1 enzymes was shown to promote a metabolite network (intra- and inter-cellular) that induces DCs immunosuppressive properties [54].

Gut microbiome can direct utilize the amino acid Trp limiting its availability to the host. This per se may affect host immune system. Besides using IDO activity to generate kynurenine or hydroxykynurenine, intestinal microorganisms can also transform Trp into several indoles that are ligands for aryl hydrocarbon receptors (AHR) [55,56]. Such ligands activate AHR, which was demonstrated to induce IDO expression in LPS stimulated DCs, promoting immune tolerance and differentiation of naïve T cells into T regulatory cells [57]. It is also known that bacteria synthesize diverse d-amino acids that are then enantioselectively recognized by some receptors and enzymes in mammals and may mediate adaptive immunity [58,59]. Synthesis of d-amino acids by bacteria involves mechanisms catalyzed by racemase, epimerase or aminotransferase enzymes, resulting in direct interconversion of the l- and d- stereoisomers [60]. In vivo, IDO can catabolize d-Trp into D-kynurenine which is further metabolized to kynurenic acid by d-amino acid oxidase (d-AAO) or kynurenine aminotransferase (KAT); or it is converted into d-3-hydroxykynurenine by kynurenine 3-monooxygenase [61]. The results presented here paved the way for our research that identified the d-form of the amino acid tryptophan, isolated from supernatants of LGG and W56 culture, as an immunomodulator in vitro and in vivo, while its l-form and other d-amino acids were inactive. Whether d-metabolites derived from d-Trp induce tolerogenic DCs or not, has yet to be discovered.

Metabolic pathways involving other amino acid also characterized the group of bioactive supernatants, especially aromatic ones such as phenylalanine and tyrosine which are suggested to be putative precursors of bioactive bacterial metabolites [62]. Another example of a pathway distinguishing the group of bioactive supernatants, which plays a crucial role in the development of immune cells (inducing immunity or tolerance) and in many autoimmune diseases, is the Aminoacyl-tRNA biosynthesis (ARS) [63]. The identification of probiotic derived metabolites belonging to theses pathways and the assessment of their potential as immunomodulators, is a stimulus for future studies. Overall, the advantage of the FT-ICR-MS metabolomics-based supernatant screening strategy for our probiotic research is that predicted hits (or identified bioactive compound candidates) are often commercially available or are easy to synthetize and can be directly tested in bioassays prior or without laborious bioassay-guided fractionation investigation.

### 3.3. Implications and Limitations of the Study

To reasonably compare the supernatants samples using FT-ICR-MS spectra, differences in cell culture had to be as limited as possible, as well as the number of medium components that could potentially cause MS detection interferences. As such, a simple chemically defined medium (CDM1) was preferred to cultivate all 25 probiotic strains under the same conditions whilst recognizing the fact that some strains initially available did not grow adequately in this minimal essential medium and had to be excluded from the screening experiment, and also the fact that a full growth medium with various sources of compounds could potentially spark a different metabolic footprint if it was applied [64]. On the other hand, each of the 25 probiotic supernatants harvested from CDM1 medium showed a concordant immunomodulatory response with supernatants harvested from the same strains cultivated in complex de Man Rogosa-Sharpe (MRS) medium [30,31].

Centrifugation followed by fast filtration was a quick and simple method to harvest cell-free supernatants (aliquots of supernatants were incubated after that and no cell growth was observed proving that those supernatants were cell-free). The loss of certain set of metabolites during this procedure due to, for example, binding to the filter material or lack of stability was not investigated, as well as leakage of bacteria cells intracellular metabolites into the supernatant. However, further investigation of the drawbacks of the fast filtration method was beyond the scope of this study once immunomodulatory activity could be proven in the harvest of cell-free supernatants thereafter (i.e., the compound/s responsible for the immunomodulatory effect in our bioassay was/were present in the collected supernatant sample).

The loss of metabolites during sample preparation using SPEs may not be excluded as well. It is possible that some metabolites were not retained in the solid phase during the first SPE step (sample load) or subsequently washed out during the washing step. Only the eluted samples (MeOH extract) were analyzed by FT-ICR-MS. To overcome that, aliquots of bacteria supernatants were taken crude and analyzed without being subjected to SPE extraction, which unfortunately did not generate a robust separation in OPLS.

FT-ICR-MS analysis by direct infusion enabled us to rapidly screen the probiotic stains of highest bioactive activity, which led to important pathway information. This analytical approach did not enable isomer’s differentiation, but neither a classical non-targeted LC/MS method would have been able to achieve that. Due to the well-known importance of stereochemistry in biological processes, follow-up in vitro assays to test isomers activity and sample analysis using complementary techniques such as enantioselective chromatography and NMR was essential to distinguish D-tryptophan as a chiral molecule within the most bioactive compound candidates in our follow-up study.

## 4. Materials and Methods

### 4.1. Probiotic Strains Cultivation and Supernatant Collection

Shortly, a total of 20 probiotic strains were obtained from WINCLOVE Probiotics B.V. (Amsterdam, The Netherlands) whereas 5 other ones were purchased from three different sources (Table 1). As already described in our former work [31], strains were cultivated separated using chemically defined medium CDM1 [65] without Tween^®^ 80 as it is known to cause interferences in mass spectrometry analyses [66]. Cells were growth under sterile microaerobic/anaerobic conditions at 37 °C and bacteria concentration was followed by optical density of the medium (OD) at 600 nm. Supernatants were collected only after cells had reached stationary phase and a minimum cell number of 10^8^ colony-forming units/mL. Fast filtration method was applied to separate cells from medium and cell-free supernatants were stored at −20 °C right after filtration and then at −80 °C until analysis. A portion of each supernatant was saved for bioassay screening as well.

### 4.2. Bioassays for Immunomodulatory Activity in Probiotic Supernatants

Results of two biological assays previously performed were used in this study to assign probiotic supernatants into groups. The bioactivity screenings were based on probiotic supernatants ability decrease the CC chemokine ligand 17 (CCL17) secretion in a human Hodgkin lymphoma cell line (approx. 70% decrease relative to untreated cells and to supernatant from the non-probiotic *Lacticaseibacillus rhamnosus* DSM-20021; negative control), and to concordantly prevent upregulation of costimulatory molecules in LPS stimulated human dendritic cells, both assays are described in detail elsewhere [31].

### 4.3. Sample Pre-Treatment Prior to Chemical Analysis

Probiotic strains were cultivated in water-based and highly salted media, which may also contain lipids and proteins produced by the bacteria. These are potential interferes that must be removed from the samples prior to MS analysis. Thus, Oasis^®^ HLB SPE cartridges (100 mg, 1 mL; Waters, Milford, MA, USA) and Bond Elut™ Cyano (CN-E) SPE cartridges (100 mg, 1 mL, Agilent, Inc., Walnut creek, CA, USA) were first conditioned with 1 mL methanol and then equilibrated with 1 mL of water each. Cell-free culture supernatant of each strain and sterile CDM1 medium (Blank) were treated as follows: A portion of 5 mL were first centrifuged at 5000 rpm for 10 min at 10 °C, 2 mL of the upper layer were carefully taken and submitted to HLB-SPE and 2 mL to CN-E SPE purification. Each cartridge was then cleaned with 1 mL water and metabolites were eluted with 1 mL MeOH. Methanolic extracts were stored at −80 °C until analysis. It was also of interest to analyze crude supernatants as well (i.e., without being submitted to SPE extraction). Thus, 2 mL of each strain cell-free supernatant were transferred to Falcon™ tubes and were subject to protein precipitation with 4 mL cold acetonitrile (previously stored at −20 °C) during 20 min in ice bath immersion. Then, solutions were centrifuged at 11,000 rpm for 10 min at 10 °C and the upper layer of each sample were carefully taken and stored until analysis.

All solvents and water were LC-MS grade quality (CROMASOLVE^®^, Fluka^®^ Analytical, Sigma-Aldrich-Aldrich, St. Louis, MO, USA).

### 4.4. FT-ICR-MS Chemical Analyses

Ultrahigh resolution mass spectra were acquired on a Fourier Transform Ion Cyclotron Resonance Mass Spectrometry (SolarixTM, Bruker Daltonics GmbH, Bremen, Germany) equipped with a 12 Tesla super conducting magnet (magnex scientific Inc., Yarnton, GB, UK). An Apollo II electrospray source (Bruker Daltonics GmbH, Bremen, Germany) was used for ionization. Prior to sample analysis, the instrument was calibrated with on arginine clusters (*m*/*z* 173.10440, 347.21607, 521.32775 and 695.43943) by injecting 1 ppm arginine solution. Calibration errors in this mass range were below 0.1 ppm.

SPE extracted samples were diluted 1:10 (v:v) for ESI positive mode analyses and 1:50 for negative mode. Non-extracted supernatants were diluted 1:10 for both ESI positive and negative mode analyses. Dilutions were done with 70% MeOH aqueous solution containing 0.1% FA and these dilution factors were defined after preliminary MS detection testes. Supernatant extracts were injected with a Gilson autosampler (223 Sample Changer, Gilson Inc., Middleton, OH, USA) where samples were kept cooled at 8 °C during the analysis. Samples were analyzed by blocks of sample types: HLB extracts, CN-E extracts, and crude supernatants. The sample analysis order was randomized between bioactive and non-bioactive supernatants inside each block. After the analysis of a group of 10 samples, three spectra of pure methanol were acquired to minimize cross-contamination and ion memory effects. MS data were acquired using the program ApexControl 1.5 (Bruker Daltonics) and the instrument has been tuned to obtain highest sensitivity for metabolite detection in each type of sample in a 10 min run time (Table S1).

### 4.5. Data Processing and Data Analysis

Data processing of FT-ICR-MS spectra is an important step to originate meaningful data matrices used in multivariate statistical analyses and data interpretation. The spectra have been extracted and processed using the software DataAnalysis 4.0© (Bruker Daltonics GmbH, Bremen, Germany). Internal calibration of positive mode spectra was done according to a mass list of endogenous abundant metabolites such as amino acids and cell-growth medium components, where spectra acquired in negative mode were internally calibrated with a reference mass list of fatty acids. The mass lists files (.asc) were exported with a signal-to noise ratio (S/N) of 4 for ESI[+] and 3 for ESI[-] spectra, and were aligned within a 1 ppm window with the software Matrix Generator 0.4, an in-house written software [67]. Further mass filtering was performed in each data matrix and the masses occurred in less than 10% of samples (of each class) were excluded from further analysis. Sterile CDM1 medium samples and pure solvents spectra were equally treated. Possible elemental formulas were assigned for each peak in batch mode using in-house software (FormulaCalculator) [68]. The formulae generated were validated by setting chemical constraints (N rule, O/C ratio ≤ 1, H/C ≤ 2n+2, elements count: C0-100, H0-∞, O0-80, N0-7, S0-1) in conjunction with an automated theoretical isotope pattern comparison [69,70]. The different datasets were analyzed by multivariate data analysis. The data were scaled using the unit variance method. The first model, PCA (principal component analysis), gave an overview of the main relations between samples and variables, without any prior information on the diverse classes. The models revealed the first separation between the molecular signatures. For each model we presented the amount of variance explained by the first two components R^2^Y(cumulative). A discriminatory strategy, using orthogonal partial least squares discriminant analysis (OPLS-DA), was then applied to all data matrices. The classification method drove the separation between bioactive and non-bioactive samples, increasing the likelihood of producing biologically relevant results. The lists of the most important masses were listed choosing the highest loadings values. The goodness of the fit and of the prediction were evaluated with the R^2^Y and Q^2^ values. To exclude overfitting, we computed the *p*-value of the cross-validation analysis of variance (CV-ANOVA). All the multivariate modelling has been done in SIMCA-P© 13 (Umetrics, Umea, Sweden). Discriminant mass features that originated OPLS DA models were uploaded into MetaboAnalyst web-based platform with a search range of 1.0 ppm for metabolic pathway analysis [37].

## 5. Conclusions

Although probiotic strains were cultivated in a closed and controlled environment and immunomodulation was tested in vitro—which may not reflect the in vivo conditions in the human body—untargeted analysis using direct infusion FT-ICR-MS followed by multivariate statistical analysis enabled the distinction between the group of immunomodulatory supernatants and inactive ones offering a broad overview into possible secreted bioactive compounds and the most significant metabolic pathways that differentiates the two classes of supernatants.

The results suggest that tryptophan metabolism may play an important role in regulating DCs immune tolerance and can be used to foster follow-up experiments. For example, discriminant hits can be seen as potential candidate compounds to undergo bioassay screenings which may accelerate the identification of bioactive metabolites or at least, give scientists an indication of potential bioactive classes of compounds to be explored next. This does not apply only to Trp metabolism but also to other discriminant pathways yet to be explored. Often, metabolites hits are commercially available or are easy to synthetize, but their immunomodulatory properties have been not investigated.

The findings and strategy presented here can support the selection of the most appropriate strains that should go through a more expensive and complex animal or/and human study posteriorly. It may help scientists and manufactures to better design probiotic cocktail products tailored to a special treatment and disease, and to improve interactions with regulatory agencies based on the characterization of strains metabolic footprinting. The emerging trend of postbiotics is another field that can benefit from the research approach described in this work. Studies using probiotic-derived bioactive metabolites may overcome obstacles when, for example, oral administration of probiotics is not feasible, or a specific therapeutic dose of a metabolite is needed.

## Figures and Tables

**Table 1:** Probiotic strain supernatants subjected to metabolite screening and their respective immunomodulatory response previously observed in vitro.

Bacterial Strain.	Code	Source	Effect on DC ^a^	Effect on KM-H2 ^b^
*Bifidobacterium animalis subsp.lactis*	BB-12 ^c^	Chr. Hansen, Horsholm, Denmark	+	+
*Bifidobacterium animalis*	W53	Winclove Bioindustries BV, The Netherlands	−	−
*Bifidobacterium breve*	W9	Winclove Bioindustries BV, The Netherlands	+	+
*Bifidobacterium breve*	W25	Winclove Bioindustries BV, The Netherlands	−	−
*Bifidobacterium lactis*	BB-420	Danisco, Niebüll, Germany	+	+
*Bifidobacterium lactis*	W51	Winclove Bioindustries BV, The Netherlands	−	−
*Enterococcus faecium*	W54	Winclove Bioindustries BV, The Netherlands	−	−
*Lactobacillus acidophilus*	LA-5	Chr. Hansen, Horsholm, Denmark	+	+
*Lactobacillus acidophilus*	W12	Winclove Bioindustries BV, The Netherlands	−	−
*Lactobacillus acidophilus*	W33	Winclove Bioindustries BV, The Netherlands	+	+
*Lactobacillus acidophilus*	W74	Winclove Bioindustries BV, The Netherlands	−	−
*Lacticaseibacillus casei*	LC-01	Chr. Hansen, Horsholm, Denmark	+	+
*Lacticaseibacillus casei*	W20	Winclove Bioindustries BV, The Netherlands	−	−
*Lacticaseibacillus casei*	W56	Winclove Bioindustries BV, The Netherlands	+	+
*Lacticaseibacillus casei*	W79	Winclove Bioindustries BV, The Netherlands	+	+
*Lactobacillus helveticus*	W60	Winclove Bioindustries BV, The Netherlands	−	−
*Lacticaseibacillus paracasei*	W7	Winclove Bioindustries BV, The Netherlands	+	+
*Lactiplantibacillus plantarum*	W21	Winclove Bioindustries BV, The Netherlands	−	−
*Lactiplantibacillus plantarum*	W62	Winclove Bioindustries BV, The Netherlands	−	−
*Lacticaseibacillus rhamnosus*	LGG	Valio Ltd., Helsinki, Finland	+	+
*Lacticaseibacillus rhamnosus*	W102	Winclove Bioindustries BV, The Netherlands	−	−
*Ligilactobacillus salivarius*	W24	Winclove Bioindustries BV, The Netherlands	−	−
*Lactococcis lactis*	W32	Winclove Bioindustries BV, The Netherlands	−	−
*Streptococcus salivarius*	W122	Winclove Bioindustries BV, The Netherlands	−	−
*Streptococcus thermophilus*	W69	Winclove Bioindustries BV, The Netherlands	−	−

^a^ Ability to decrease the percentages of human-derived dendritic cells (DCs) expressing costimulatory molecules CD83-, CD80-, CD86-, and CD40 after lipopolysaccharide (LPS)-induced maturation, by at least 30% relative to untreated DCs and DCs treated with supernatant from the non-probiotic *Lacticaseibacillus rhamnosus* DSM-20021 (negative controls). ^b^ Ability to lower CCL17 secretion by KM-H2 cells in a dose-dependent manner to concentrations of approx. 30% of those observed in untreated cells and in cells treated with supernatant of the non-probiotic *Lacticaseibacillus rhamnosus* DSM-20021 (negative controls). ^c^ Apart from BB-12, probiotics providers did not disclose further details on subspecies.

**Table 2:** Number of *m*/*z* features present in each data matrix after data processing and molecular formula annotation.

ESI Mode	Sample Pretreatment Applied		
	Crude Supernatant *	HLB-SPE Extract	CN-E-SPE Extract
Positive **	344	2658	1367
Negative	1970	3932	3353

* Crude supernatant was subject to protein precipitation with cold acetonitrile, followed by centrifugation (the upper layer of each sample was carefully taken and diluted prior analysis). No solid phase extraction was applied. ** Signal-to noise ratio (S/N) of 4 was applied as substantial amount of noise signals was not excluded with S/N 3. The significant lower amount of *m*/*z* annotated features presented in crude-supernatant analysis compared to SPE-extracts can be a result of ion suppression caused by growth medium components at high concentration; while SPE extracts concentrate metabolites and remove, to a certain extent, medium ingredients from the sample.

**Table 3:** Tryptophan metabolism pathway activity prediction (hits) directly from mass peaks of the most discriminant features (KEGG compound codes).

Bioactive Supernatants SPE Extract			Non-Bioactive Supernatants Extract	Metabolite Hit Prediction *
HLB-SPE/ESI[+]	HLB-SPE/ESI[-]	CN-E-SPE/ESI[-]	HLB-SPE/ESI[-]	
C00078	C00078	NP	NP	Tryptophan
C00331	C00331	C00331	NP	Indolepyruvic acid
C00643	C00643	C00643	C00643	5-Hydroxy-l-tryptophan
C01598	C01598	NP	C01598	Melatonin
C00978	C00978	NP	NP	N-Acetylserotonin
C00780	C00780	C00780	NP	Serotonin
C02298	C02298	C02298	C02298	N-Acetylindoxyl
C02700	C02700	C02700	C02700	l-Formylkynurenine
C00328	C00328	C00328	NP	l-Kynurenine
C03227	C03227	NP	NP	3-Hydroxy-l-kynurenine
C00637	C00637	C00637	C00637	Indole-3-acetaldehyde
C02693	C02693	NP	NP	Indole-3-acetamide
C00954	C00954	C00954	C00954	Indole-3-acetic acid
C02937	C02937	NP	NP	Indole-3-acetaldehyde oxime
C03230	C03230	C03230	C03230	3-Indoleglycolaldehyde
C02043	C02043	NP	C02043	Indolelactate
NP	C00955	NP	C00955	Indole-3-ethanol
		C02470		Xanthurenic acid
	C01987			2-Aminophenol
	C01249			7,8-Dihydro-7,8-dihydroxykynurenate
	C01717			Kynurenic acid
	C00398			Tryptamine
	C02172			N-Acetylisatin
	C01252			4-(2-Aminophenyl)-2,4-dioxobutanoate
	C00463			Indole; 2,3-Benzopyrrole
			C02775	Dihydroxyindole
			C02938	3-Indoleacetonitrile
			C03574	2-Formylaminobenzaldehyde

* Metabolites are not structurally elucidated by mean of FT-ICR-MS but displayed as hit prediction based on high accuracy mass (<1.0 ppm mass error). NP: Not present.

## Data Availability

Data, tables and figures are contained within the article and Supplementary Material.

## Supplementary Materials

The following are available online at https://www.mdpi.com/article/10.3390/metabo12010035/s1, Figure S1: A comparison visualization using Venn diagrams, Table S1: Instrumental parameters used for the analyses of cell-free supernatant extracts. Single MS acquisition mode and ESI ionization.
