# Supplementary material for: Mining for Active Molecules in Probiotic Supernatant by Combining Non-Targeted Metabolomics and Immunoregulation Testing
Source: Metabolites. 2022 Jan 4;12(1):35. doi: 10.3390/metabo12010035 (PMC8778235; doi:10.3390/metabo12010035)
Supplement: Supplementary file 1 [file metabolites-12-00035-s001.zip › Supplementary_Table_1.pdf]

Table S1: Instrumental parameters used for the analyses of cell-free supernatant extracts. Single MS acquisition mode and ESI ionization.

Parameters	(-) FT-ICR-MS	(+) FT-ICR-MS
HLB and CN-E extracts		
Mass range	147.4 – 2000 Da	147.4 – 1500 Da
Source accumulation	0.01 sec	0.05 sec
Ion accumulation time	0.30 sec	0.50 sec
Acquired scans	500	300
Capillary voltage	4000 V	4000 V
Drying gas flow rate	4.0 L/min	4.0 L/min
Drying gas temperature	180 °C	200 °C
Nebulizer gas flow rate	1.0 bar	1.0 bar
Spray shield	-500 V	-500 V
Excitation pulse time	5.0 µsec	5.0 µsec
Analyzer entrance	4.0 V	-5.0 V
Sidekick offset	1.5 V	-1.5 V
Trap	-20 V	20 V
Front trap plate	-0.4 V	0.4 V
Back trap plate	-0.5 V	0.5 V
Transient time domain	2 MW	2 MW
Syringe flow rate	120 µL/h	240 µL/h
Run time	10.0 min	10.0 min
Crude supernatants		
Mass range	73.7 – 1000 Da	73.7 – 1000 Da
Source accumulation	0.01 sec	0.01 sec
Ion accumulation time	0.30 sec	0.30 sec
Acquired scans	500	400
Capillary voltage	3600 V	3600 V
Drying gas flow rate	4.0 L/min	4.0 L/min
Drying gas temperature	180 °C	180 °C
Nebulizer gas flow rate	2.0 bar	2.0 bar
Spray shield	-500 V	-500 V
Excitation pulse time	15.0 µsec	15.0 µsec
Analyzer entrance	4.0 V	-10.0 V
Sidekick offset	10.0 V	-1.5 V
Trap	-20 V	20 V
Front trap plate	-0.4 V	0.4 V
Back trap plate	-0.5 V	0.5 V
Transient time domain	2 MW	2 MW
Syringe flow rate	120 µL/h	120 µL/h
Run time	10.0 min	10.0 min
