# Supplementary figures and images for: Mining for Active Molecules in Probiotic Supernatant by Combining Non-Targeted Metabolomics and Immunoregulation Testing
Source: Metabolites. 2022 Jan 4;12(1):35. doi: 10.3390/metabo12010035 (PMC8778235; doi:10.3390/metabo12010035)

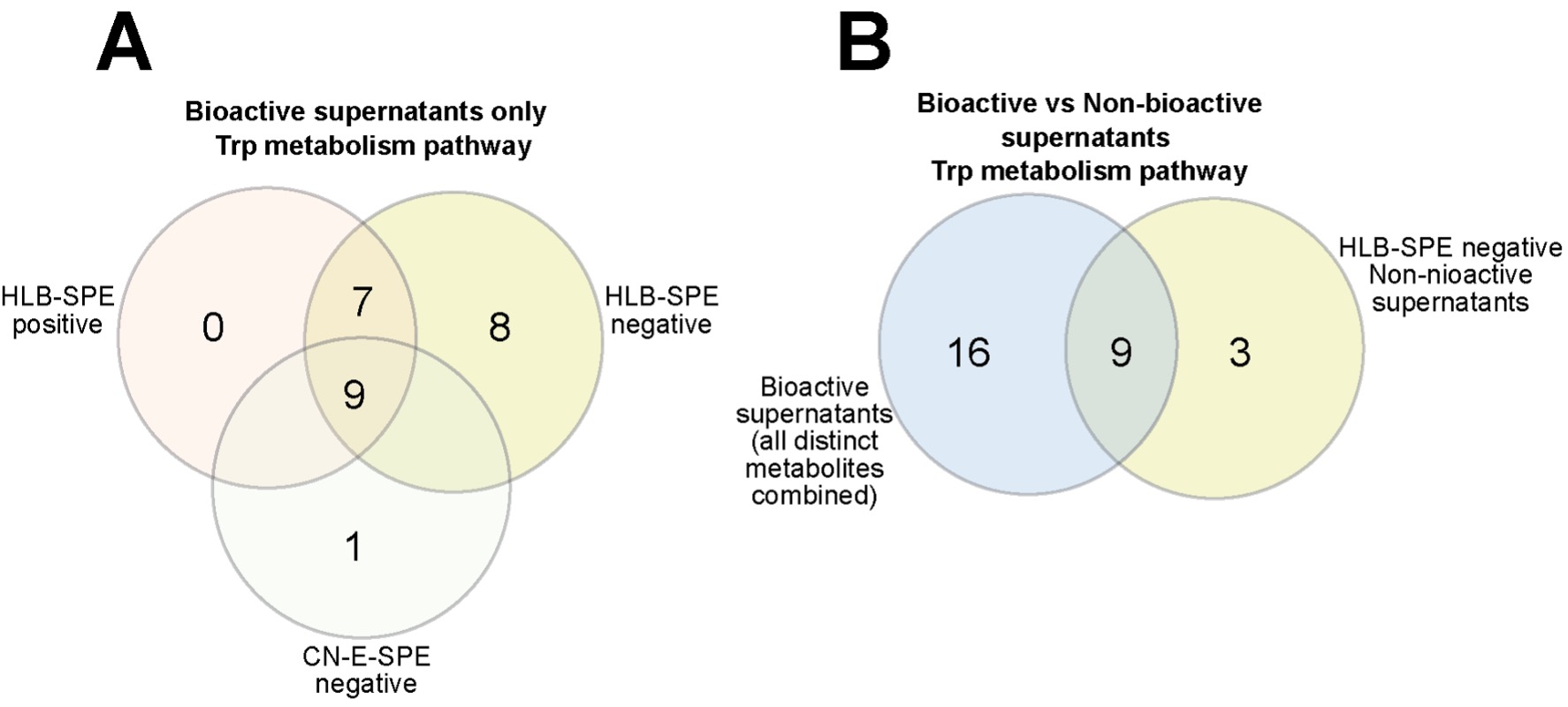

Supplement: Supplementary file 1 [file metabolites-12-00035-s001.zip › Supplementary_Figure_1.jpg]
